# Silver Nanoparticle’s Toxicological Effects and Phytoremediation

**DOI:** 10.3390/nano11092164

**Published:** 2021-08-24

**Authors:** Muhammad Ihtisham, Azam Noori, Saurabh Yadav, Mohammad Sarraf, Pragati Kumari, Marian Brestic, Muhammad Imran, Fuxing Jiang, Xiaojun Yan, Anshu Rastogi

**Affiliations:** 1 College of Landscape Architecture, Sichuan Agricultural University, Chengdu 611130, China; ihtisham@sicau.edu.cn (M.I.); bnjfx@sicau.edu.cn (F.J.); 2 Department of Biology, Merrimack College, North Andover, MA 01845, USA; nooria@merrimack.edu; 3 Department of Biotechnology, Hemvati Nandan Bahuguna Garhwal (Central) University, Garhwal, Srinagar 246174, Uttarakhand, India; saurabhyadav40@rediffmail.com; 4 Department of Horticulture Science, Shiraz Branch, Islamic Azad University, Shiraz 71987-74731, Iran; sarraf.science@gmail.com; 5 Scientist Hostel-S-02, Chauras Campus, Garhwal, Srinagar 246174, Uttarakhand, India; pragati27@gmail.com; 6 Department of Plant Physiology, Slovak University of Agriculture, A. Hlinku 2, 94976 Nitra, Slovakia; marian.brestic@uniag.sk; 7 Department of Botany and Plant Physiology, Faculty of Agrobiology, Food and Natural Resources, Czech University of Life Sciences Prague, 16500 Prague, Czech Republic; 8 State Key Laboratory for Conservation and Utilization of Subtropical Agro-Bioresources, College of Agriculture, South China Agricultural University, Guangzhou 510642, China; muhammadimran@scau.edu.cn; 9 Laboratory of Bioclimatology, Department of Ecology and Environmental Protection, Poznan University of Life Sciences, Piątkowska 94, 60-649 Poznan, Poland; 10 Faculty of Geo-Information Science and Earth Observation (ITC), University of Twente, 7500 AE Enschede, The Netherlands

**Keywords:** phytoremediation, silver nanoparticles, toxicological effects, environmental sustainability

## Abstract

The advancement in nanotechnology has brought numerous benefits for humans in diverse areas including industry, medicine, and agriculture. The demand in the application of nanomaterials can result in the release of these anthropogenic materials into soil and water that can potentially harm the environment by affecting water and soil properties (e.g., soil texture, pH, organic matter, and water content), plants, animals, and subsequently human health. The properties of nanoparticles including their size, surface area, and reactivity affect their fate in the environment and can potentially result in their toxicological effects in the ecosystem and on living organisms. There is extensive research on the application of nano-based materials and the consequences of their release into the environment. However, there is little information about environmentally friendly approaches for removing nanomaterials from the environment. This article provides insight into the application of silver nanoparticles (AgNPs), as one of the most commonly used nanomaterials, their toxicological effects, their impacts on plants and microorganisms, and briefly reviews the possibility of remediation of these metabolites using phytotechnology approaches. This article provides invaluable information to better understand the fate of nanomaterials in the environment and strategies in removing them from the environment.

## 1. Introduction

Nanotechnology is a growing and advanced area of science and engineering that focuses on the synthesis, and application of matters on the nano scale with at least one dimension less than 100 nm [1]. The characteristics of nanomaterials are mainly derived from their small size, high surface area/volume ratio, and high stability [2]. Compared with conventional materials, the properties of engineered nanomaterials have improved their application [3,4]. They have physical properties such as high uniformity, high conductivity, or special optical properties, making them popular in chemistry, physics, and biology [5].

Nanotechnology is used in various areas [6,7], including agriculture to improve crop yield by offering innovative agrochemical formulations and delivery methods, which in turn reduces the need for pesticide application [8]. Nanoparticles are also used in precision farming [9] to achieve sustainable agriculture, as these particles are attributed to altering critical plant life events and are used in agriculture for a variety of purposes such as minimizing nutritional loss, reducing various environmental stresses, and increasing crop yield [10,11]. In addition, nanoparticles are used in agriculture as nanofertilizers, nanopesticides, or nanosensors to protect plants against pathogens and improve productivity [12].

Extensive research on the impacts of nanoparticles on living organisms suggests that nanoparticles have “grey shade” by resulting in both pros and cons effects [13]. Metal-based nanoparticles are among the most recent types of anthropogenic materials that can potentially harm the ecosystem if used in a high concentration [14]. Nanoparticles can be released into the soil during production, agricultural or industrial applications, or accidental spillage [15]. Nanoparticles can also increase the bioavailability of metals in the soil and potentially result in higher environmental risk [16].

Different types of engineered NPs are synthesized for various applications. Silver nanoparticles (AgNPs) are among the most commonly used engineered nanomaterials with medicinal, industrial, and agricultural applications [17]. Considering the vast usage of AgNPs, there is a possibility of their release into the environment, and their potential toxicological effects on plants and animals. Apart from using the particulate form of silver, AgNPs may be transformed to silver oxide or silver sulfide via oxidation or sulfidation, respectively, and these ones impact the soil and living organisms in a variety of ways [18]. Therefore, it is critical to address the behavior of nanoparticles in the environment and possible methods for their removal. This review focuses on three objectives to discuss this issue including: the possible pathways for the release of AgNPs into the environment; the toxicological effects of AgNPs on plants and microorganisms; and the recommended phytoremediation approaches. This review provides invaluable information for a more sustainable application of AgNPs.

## 2. Applications of Silver Nanoparticles

The application of silver particles has a centuries-old history due to their therapeutic nature in medicine and storage vessel for beverages [19]. AgNPs are among the most commonly used nanoparticles, broadly due to their numerous uses. In 2015, the AgNPs market was reported to be $1 billion, which is expected to rise to $3 billion by 2024 [20,21]. Due to the antibacterial, antifungal, antiseptic, and antiviral properties, AgNPs are of prime importance in medical applications and were broadly used as an antimicrobial agent before the discovery of antibiotics [19,22,23]. Up until now, AgNPs have been utilized in a diverse range of applications in many fields, with some examples of health (immunity-based food supplements, pharmaceuticals, disinfectants, burn treatment products, and wound healing/dressings, radiotherapy), other biomedical products, optics, biosensing, environmental remediation, food industries, cleaning, water and air disinfection, electronics, textile, packaging, skincare products, detergents, plastics, paints, and even children toys [21,24,25,26,27]. AgNPs have also been used in agriculture as plant growth promoter/fertilizer, fruits ripening and preservation agent, plant disease control fungicide, and insecticide [25,28]. AgNPs also have several distinctive physicochemical properties, such as high thermal and electrical conductivity, improved surface Raman scattering, catalytic activity, and non-linear optical behavior [29]. This widespread use of AgNPs ensures particle release into the environment and makes it one of the most exploited nanomaterials [30,31] resulting in unknown impacts on plants, microorganisms, animals, and humans in the exposed environment.

AgNPs have the average production of 500 tons per year [32] and are known for their widespread application which has made their entry into many commercial products [33,34,35]. They have diverse applications and are becoming more common in industrial processes [36]. The high usage of AgNPs can result in the direct or indirect release of these nanomaterials into the environment and can potentially cause toxicity for various organisms including aquatic organisms, plants, and humans. The direct transformation includes aerial deposition, run-off, or sewage discharges (release from industrial and household products into water bodies). The indirect release includes discharges from organic or inorganic fertilizers and engineered plant growth substances used as fertilizer substitutes that can be accumulated in soil and underground water, accidental spills during manufacturing and transport, and biosphere pollutions through atmospheric emissions from smelting, coal combustion, and cloud seeding [37,38,39,40,41]. AgNPs can be oxidized in the environment and be transformed into the ionic form of the silver (Ag^+^) which can be more toxic than the particulate form of silver [42]. AgNPs and Ag^+^ can make their way into the water bodies and soils during or after the lifetime of the product and eventually affect the ecosystem [30,43,44].

Prior to evaluating plant’s potential to remove nanomaterials from the environment, it is critical to understand the environmental toxicology effects of these materials and their effects on plants and microorganisms.

## 3. Environmental and Toxicological Effects of AgNPs

The heavy inclusion of AgNPs in today’s world has not been assessed and correlated with environmental risks [45,46] and balance between nanoparticles and biodiversity. Recently, the application of nanomaterials has gained more popularity in different areas [47,48] which raises the risk of their release into the environment and potential toxicological effects. AgNPs are considered as enormously toxic on the basis of L(E) C_50_ values (the amount which causes 50% death of tested animals when the material was given at once) for environmentally related organisms at the levels of L(E) C50 b 0.1 mg L^−1^ [32], while on the other hand, the predicted environmental concentration (PECs) of AgNPs in the environment ranges between 0.03 to 0.08 mg L^−1^ [49]. As per the World Health Organization (WHO) and Environmental Protection Agency (EPA), the maximum contamination limit (MCL) of toxic range of silver is 0.1 mg L^−1^ [50,51,52,53,54], although naturally occurring silver concentrations are generally low in the environment (surface waters) but are continuously increasing at higher levels due to runoff and wastewater from urban and industrial areas [50]. AgNPs released into the environment can be oxidized and generate the ionic form of silver that is more reactive than the particulate form. The high concentration of AgNPs and their potential to be oxidized in the environment can cause toxicity for living organisms. Consequently, this dilemma, if not addressed on time, will more negatively affect the ecosystem also by inhibiting growth of plants, polluting drinking water, and causing harm to human health. This will ultimately curtail the fauna and flora. Therefore, sustainable methods for the remediation of contaminated soil with AgNPs must be investigated.

Since the 1980s, the U.S. Environmental Protection Agency (EPA) regulated AgNPs usage and release into the environment to limit their impacts on the ecosystem [55]. To curb their impacts on living organisms, the concentration of total silver in aquatic systems is limited to 1.2–13 ppb (depending on CaCO_3_ concentration) [55]. Over the last decade, the vast application of AgNPs called for examination of these particles to determine their long-lasting effects on ecosystems. The occurrence of silver species in the environment, particularly the water supply, can result in bioaccumulation across several trophic levels with potentially severe toxic effects [56]. Due to the increasing consumption of these materials, the aquatic environments are prone to silver contamination. The released AgNPs into the environment can be transformed into more toxic forms such as chloride, nitrate, etc. [57]. Among these silver species, silver nitrate is the most toxic due to dissociation into Ag^+^ and nitrate interaction with other elements. Therefore, it is crucial to increase our understanding about the toxicity effects of AgNPs [56].

**Figure 1:**
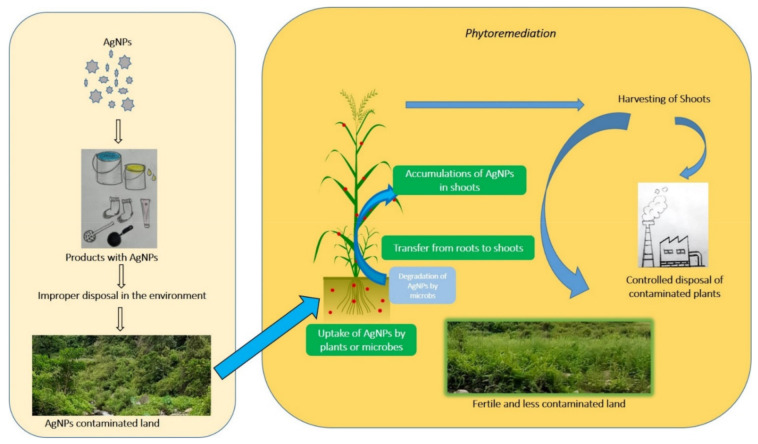
Potential pathway for AgNPs leakage into the environment and its remediation.

Silver is considered as the second most toxic metal to aquatic organisms after mercury [58]. The toxicity mechanisms of AgNPs and Ag^+^ are different but both are fatal to a variety of organisms including bacteria, animals, and plants [37,59]. The cellular structure and organelles of these organisms are affected by silver species through unfavorable binding interactions [60]. Silver cations can potentially enter cells through metal transporters such as copper ion transporters or transmembrane potassium channels [24]. Moreover, silver species translocation is attributed to variations in toxicity among Ag^+^, AgNPs, and insoluble silver salts [37,61]. The potential pathway for AgNPs released into the environment and its remediation is illustrated in Figure 1.

### 3.1. Effects of AgNPs on Plants

Unsustainable application of nanoparticles and their release into the environment can also negatively affect the economy by reducing the quality of crops and affecting human health. AgNPs released in waste or ground water can end up in plant growth environment. The small size of nanoparticles enables them to enter plant roots and travel short distances cell by cell or long distances, roots to shoots, via xylem cells [62]. AgNPs accumulated in plant tissues can subsequently enter the human body via the food chain. Numerous studies have reported the toxicological effects of AgNPs on plants (Table 1). The level of toxicity and the impact of AgNPs on plants depend on various factors namely the size, type, and concentration of nanoparticles as well as plant species, soil type, hydraulic conductivity, and the availability of essential nutrients in soil [63,64]. As small as the NPs are, their toxicity is still significant since they can be dissolved easier in water and enter living organisms [65]. As mentioned above, AgNPs have a tendency to be oxidized in the environment and form the ionic form of silver (Ag^+^). Therefore, plants exposed to AgNPs are also affected by Ag+ that is more interactive and toxic than the particulate form. Noori, et al. [21] detected both the ionic and particulate forms of silver in leaves of tomatoes (*Lycopersicon esculentum* L.) exposed to 10, 20, 30 mg L^−1^ of 20 nm AgNPs for 7 days via roots. It confirms that AgNPs can be directly taken up by plants and be stored in tissues in the form of particulates. The presence of AgNPs or Ag^+^ in cells affects cellular components, metabolism, and plant growth at the morphological, molecular, and physiological level. At the morphological level, AgNPs affect seed germination, root development, and cellular compartments [66,67]. The study by Qian et al. [68] showed that the thylakoid membrane and the structure of chloroplasts were affected in *A. thaliana* exposed to AgNPs. This impacts photosynthesis, metabolism, and plant growth rate. The study on Oryza sativa seedlings exposed to 0.5–1 mg L^−1^ of AgNPs showed significant lowering of mitochondrial membrane potential [69]. A significant decrease in the photosynthetic rate, CO_2_ assimilation, and plant growth is also reported in tomatoes exposed to 10 mg/kg of 7–14 nm AgNPs for 72 weeks [70]. Plant responses at the physiological level are modified by the expression of genes and proteins. Kaveh, et al. [71] reported the effects of AgNPs on the expression of over 300 genes. They reported the upregulation of 286 genes and downregulation of 81 genes in Arabidopsis thaliana seedlings exposed to up to 20 mg/L of 20 nm AgNPs for 10 days. Proteomics studies also show that AgNPs affect the expression of proteins involved in signal transduction, defense, and oxidative stress responses [72,73]. It implies that the effect of AgNPs on plants is related to the production of ROS and increased production of antioxidant enzymes which further affect the decrease in shoot and root growth and other pigments. This decreases photosynthesis, plant biomass and crop productivity.

In addition, several studies have reported a significant increase in the concentration of hydrogen peroxide (H_2_O_2_), hydroxyl radical (°OH), and malondialdehyde (MDA) as oxidative stress indicators upon exposure to AgNPs [81,84]. It means exposure to AgNPs can result in oxidative stress in plants. Oxidative stress increases ROS in plants under various environmental stresses, which is then countered by enzymatic and non-enzymatic molecules [85,86,87]. The level of oxidative stress and plant’s ability to cope with NPs varies based on plant species, the size of nanoparticles, and environmental conditions [64,65,80]. Plants that can tolerate NPs in their environment have higher potential to synthesize antioxidants. Antioxidative metabolites (e.g., flavonoids, anthocyanins, or phenols) and enzymes (e.g., catalase, peroxidase, and superoxide dismutase) improve plants ability to reduce the oxidative stress induced by AgNPs.

Despite the extensive research on the toxicity effects of AgNPs on plants, there are reports on induced seed germination and plant growth upon exposure to AgNPs [88,89]. This could be due to AgNPs’ role in improving water and nutrient uptake [90]. It is suggested that high concentration of NPs in the environment results in the aggregation of particles and creates larger particles (>100 nm). The aggregated particles do not easily enter plant cells and are less toxic than smaller particles [91]. On the other hand, AgNPs, as a type of oxidative stressor, can induce plant antioxidative and defense responses and improve plant tolerance in stressed condition. Kruszka et al. [92] reported upregulation of secondary metabolites involved in defense responses in *A. thaliana* exposed to 0.5–5 mg L^−1^ AgNPs. They suggested that AgNPs have a role in improving the bioavailability of nitrogen. Elevated level of antioxidant enzymes and metabolites induce plant’s ability to tolerate other environmental stresses as well. Khan et al. [93] reported that exposure to 30 mM AgNPs induced antioxidative responses of *Pennisetum glaucum* L. exposed to 150 mM NaCl. It suggests that plants with potential to tolerate AgNPs in their environment can be considered for phytotechnology approaches to simultaneously remove AgNPs and other contaminants from the environment. In summary, the effects of AgNPs on plants can be either induction or inhibition of growth, development, or defense responses based on various factors such as the type, size, concentration of AgNPs, as well as plant species and the period of exposure. The plant microbe interaction and the effects of AgNPs on microorganisms should be taken into consideration in phytotechnology approaches.

### 3.2. The Effects of AgNPs on Soil Microorganisms

The antimicrobial activities of AgNPs affect soil-borne microorganisms including pathogenic and beneficial bacteria and fungi [94,95]. The release of AgNPs in soil decreases soil microorganisms’ population [96,97] and results in higher toxicity in soil. In addition to the effects on the soil microbial community, AgNPs also affect the activity of soil microorganisms [27,98]. AgNPs can both induce or inhibit enzymatic activities of soil microbes [99,100] depending on the type of the microorganisms, soil texture, osmotic potential, size, shape, and the concentration of AgNPs [101]. Jain et al. [102] reported that the enzymatic activity of decomposing bacteria and fungi depends on the concentration of NPs. They reported that exposure to 2.5 µM AgNPs induced the enzymatic activity of several fungi and bacteria, while 25 and 50 µM exposure had the opposite effect Tripathi, et al. [103] summarized the effects of AgNPs on soil microorganisms. They highlighted that AgNPs and Ag^+^ affect cell membrane integrity and enter the bacterial cell by disrupting the structure of the cell membrane. The impact on the membrane is the key in the antibacterial properties of AgNPs [104]. Exposure to AgNPs also interrupts the activity of membrane transporters and the transportation of essential elements in cells such as potassium and chloride. AgNPs and Ag^+^ entered bacterial cells interfere with DNA replication [105] and bacterial growth. Based on recent studies, it is suggested that the antibacterial properties of AgNPs are mostly related to their impacts on the structure of membrane and cellular division. Similar to plants and bacteria, other studies on the interaction of AgNPs and fungi suggest that the size of AgNPs play a vital role in this interaction [25,103,106]. The presence of chitin gives a semipermeable structure to the fungal cell wall and reduces the penetration of larger size AgNPs into fungal cells [107]. In addition to soil bacteria and fungi, AgNPs can also affect decomposers. The study on plant decomposing invertebrate *Limnephilus* sp. showed that the leaf shredding behavior of this organism is affected by the size and concentration of AgNPs [108]. Invertebrates play a vital role in soil structure and are important in inducing plant defense responses. It is important to note that changes in soil microbial population and activity affect soil properties and subsequently plant growth, physiological, and molecular responses. These impact the plant’s potential to remove nanoparticles from the environment. Table 2 lists the impacts of AgNPs on soil microorganisms.

The consequences of the release of AgNPs in the environment and their toxicological effects on organisms in their vicinity provokes the need to consider sustainable approaches to reduce their harmful effects on living organisms. Phytotechnology, the application of plants in removing or sequestering pollutants, is a promising and environmentally friendly method that can be applied in environments contaminated with NPs.

## 4. Nanoparticle’s Phytotechnology

Many soil remediation technologies (physical and chemical methods) have been established to reduce soil contamination [127,128]. However, most of these methods are laborious, time-consuming, and costly. Therefore, such technologies are not best for combating environmental pollution and remediation strategies for a long period [127]. Phytoremediation is an alternate technology applied for the remediation of pollutants using plants [129]. Plants being indispensable elements of ecosystems play a crucial role in the uptake, accumulation, and transport of elements including metal-based nanoparticles [130].

Phytotechnology refers to a technology that uses plants to remove, uptake, absorb, transform, transfer, attenuate, accumulate, degrade, or metabolite organic, inorganic, metallic, or metalloid contaminants from soil, water, or air [131,132,133,134,135]. Phytotechnology consists of several sub-methods such as rhizosphere biodegradation, phytoextraction, phytosequestration, phytovolatilization, phytodegradation, or phytoremediation. Although the phrase phytotechnology is used interchangeably with phytoremediation, the latter generally refers to a method of phytotechnology that removes pollutants from the environment. Phytoremediation is a natural, simple, cost effective, and widespread bioremediation technology that works on the principle of plant’s metabolic system to clean, recover, and remediate contaminated sites by storing pollutants in plant biomass to ensure environmental safety [129,136]. Phytoremediation strategies [136] are namely (a) phytostabilization—when plants reduce bioavailability of pollutants in soil (b) phytovolatilization—when pollutants are converted as volatile compounds by plants (c) phytoextraction—when plants take out pollutants from soil (d) phytofiltration—when cultured plants absorb pollutants from water or waste materials. There are many advantages of such phytoremediation strategies, viz. phytostabilization temporarily counter the hazardous materials as compared to phytoextraction which is a permanent one, phytovolatilization converts to gaseous compounds and is more suitable [136].

In comparison to other methods, phytoremediation provides aesthetic appearance, less destruction, and high public acceptance [137,138,139,140,141]. Since the 1980s, phytoremediation has been significantly studied, practiced, and used in field studies at contaminated sites with heavy metals, metalloids, radionuclides, oil spills, fertilizers, pesticides, chlorinated solvents, and explosives [142,143]. In Table 3, we have showed some examples of AgNPs phytoremediation description and its effects. 

Plant remediation technologies are recommended as a viable option for maintaining environmental sustainability among the various options for repairing these pollutants [148]. Recent advances in phytoremediation, molecular and metabolic engineering and nanotechnology have opened up new avenues for the effective handling of emerging organic and inorganic contaminants [149,150,151]. Plants with high potential to take up contaminants and store them in their tissues are considered as “hyperaccumulators”. These hyperaccumulators accumulate metals or metalloids at a greater level than other plants. These hyperaccumulators are found in metalliferous soils rich in any particular metals [152]. Some examples are, *Thlaspi caerulescens* [153], *Arabidopsis halleri* [154], *Pteris vittata* [155] *Pteris vittata* accumulates arsenic and three others namely *Thlaspi rotundifolium*, *Thlaspi ochroleucum*, *Thlaspi goesingese* accumulates zinc, lead and nickel, respectively [156]. There is a freely available global database containing information about hyperaccumulators (www.hyperaccumulators.org, accessed on 20 July 2021) [152].

These types of plants are favorable in phytoremediation studies especially in sites contaminated with heavy metals. In addition to heavy metals, most hyperaccumulators are also able to tolerate pollutants such as petroleum-based contaminants, explosives, or pesticides that contain a variety of heavy metals and nanoparticles [157]. Among different types of plants used in phytoremediation techniques, hyperaccumulators are best used in phytoextraction projects. AgNPs are metal based nanoparticles that can be extracted from the environment and be taken up by hyperaccumulators. To successfully implant a phytoremediation project, understanding the mechanism of uptake and translocation of contaminants, in this case metal-based nanomaterials, is required. Many studies by different groups have tried to understand the mechanism of uptake and translocation of metal NPs in plants [4,158], where the authors have indicated the size and chemical properties including zeta potential to be an important factor for initial penetration of NPs to the plant. Once the NPs enter the plants they may move through endocytosis or through symplastic transport to different plant tissues [158]. Understanding the molecular mechanism of hyperaccumulators enables scientists to use genetic engineering approaches to strengthen the remediation of environmental pollutants [159] including metal-based nanoparticles. Biotechnology is used to manipulate the expression of genes to improve hyperaccumulators potential to remove contaminants from the environment. In addition to hyperaccumulators, some plants that do not usually have the potential to remove contaminants can alter their physiology and biochemistry to release metabolites and hormones into their environment to sequester nanomaterials and reduce their reactivity in the environment [160]. This method is known as phytosequestration. To apply phytotechnology approaches in removing or sequestering NPs from the environment, several factors such as the phytotoxicity effects of NPs, plant species, weathering, soil structure and organic matter, soil microorganisms, and interaction with other chemicals should be considered [161]. The interaction of engineered NPs with soil organic matter can result in a more stable form of NPs and subsequently result in the release of the ionic form of NPs that are more interactive than the particulate form and have a higher chance to enter living organisms [161]. In addition, the hydrophobic properties of engineered NPs affect their interaction with organic matter and living organisms facilitating their uptake by plants [162]. In addition to above-mentioned factors, the chemical properties of NPs, their concentration, the ratio between the ionic and the particulate form of metal-based nanomaterials impact the phytotoxicity effects of engineered NPs and plants, potentially removing them from the environment. The study on the potential of salt marsh plant, *Phragmites australis*, in removing AgNPs and Ag^+^ showed that both AgNPs and Ag^+^ were accumulated in plant roots [49]. They emphasized that AgNPs interaction with the soil microbial community can interfere with the phytoremediation of Ag in either particulate or ionic form. Yang, et al. [163] also reported the accumulation of Ag in roots of rice exposed to up to 20 mg L^−1^ AgNPs and AgNO_3_ for five days. This study showed that uptake of AgNPs was more efficient than Ag^+^. They suggested that the high reactivity of Ag^+^ in the growth environment and generation of AgCl resulted in its lower uptake rate compared with the particulate form of Ag. The researcher has also found the accumulation of AgNPs in *Lemna gibba* after its exposure to AgNPs at a concentration of 0.01 to 10 mg L^−1^ for 7 days [164]. The authors have observed an accumulation from 7.7 to 17.5 μg/mg of AgNPs/plant dry mass. Considering the characteristics of engineered nanoparticles, it is suggested that phytoextraction and phytosequestration methods are promising approaches of phytoremediation in removing AgNPs from the environment [51,165]. The toxicity of AgNPs to plants and associated microorganisms is an important factor which is needed to be considered while the selection of plants for phytoremediation purposes. Along with the phytotoxicity effects of AgNPs and plant’s potential to remove them from the environment, various environmental and biological factors should be also considered in selecting phytotechnology as an environmentally friendly method to remove these metal-based NPs from the environment. Due to the colloidal and dynamic properties of AgNPs, rhizosphere biodegradation can prove to be an important method where the plants release certain compounds which can lead to an enhancement in microbial activity resulting in the degradation of NPs. For example, *Bacillus subtilis* can colonize the rhizosphere and get nutrition from plants. In return, it provides several benefits to plants discussed in Hashem et al. [166]. It has been reported that during wastewater treatment, the AgNPs can be partially or fully sulfidized which results in much less toxic compounds than AgNPs in its original form [167]. The authors achieved a full sulfidization with the application of *B. subtilis* indicating an important role of *B. subtilis* in the processing of AgNPs to its less toxic form. Still, the AgNPs contamination has not been identified as an immediate threat to agriculture and only a few studies have been performed which are discussed above. But the increasing use of AgNPs in day-to-day life is making it a potential threat in the near future. Thus, more investigation is needed to better use the plants as a tool to extract/destroy the AgNPs from the agricultural field.

## 5. Conclusions

This review provides invaluable information about the consequences of the release of nanomaterials in the environment. Nanomaterials, especially AgNPs affect soil properties, microorganisms, and plants and can therefore cause toxicity for living organisms including humans. Although phytoextraction is suggested as a promising approach in removing metal-based nanomaterials, several factors such as the size, concentration, and type of AgNPs as well as soil structure, soil microbial community, and plant species should be considered to select the appropriate method of phytoremediation. In addition, molecular and physiological analysis that improves understanding about the involved transporters and metabolites in removing AgNPs from the environment will help scientists to improve the success of phytoremediation of these materials by altering the expression of related genes using plant biotechnology methods.

Despite the growth in biotechnology, phytotechnology, and nanotechnology, there is still very scarce information regarding sustainable removal of toxic nanoparticles from the environment. Further research is recommended to better understand the mechanism of AgNPs uptake by plants. The scientists must evaluate a small modeling kind of approach to see the effects on ecosystem and also ‘lab to land’ approach where there will be evaluation of small-scale toxicological data and its removal must be correlated at field level. Another area must be related to seeing the molecular changes upon nanoparticle exposure. Few of these efforts will be helpful in impact assessment of nanoparticles on biological organisms.

## Figures and Tables

**Table 1:** The effects of AgNPs on plants.

Species	Size (Diameter in nm)	Concentration mg L^−1^	Impacts	References
*Solanum tuberosum* L.	20	2, 10, 20	Increase in superoxide anion (O_2_¯) and reactive oxygen species (ROS); Significant induction in the activities of superoxide dismutase (SOD), ascorbate peroxidase (APX), glutathione reductase (GR), and catalase (CAT)	[74]
*Oryza sativa* L.	20	0.2, 0.5, 1	Significant decrease in fresh weights, root elongation, shoot and root, carotenoids contents, total chlorophyll; Increased level of hydrogen peroxide (H_2_O_2_) and lipid peroxidation (MDA) in shoots and roots, increased foliar proline accumulation, and decreased sugar contents	[69]
*Wolffia globosa*	10	1, 2, 5, 8, 10	Elevated level of malondialdehyde (MDA), ROS content, and SOD activity. Decrease in chlorophyll a, carotenoids, and soluble protein	[75]
*Lycopersicon esculentum*	10–15	100, 1000	Reduced in fruits productivity, significant decreases in root growth, chlorophyll contents, and increased activity of higher SOD content	[76]
*Lycopersicon esculentum*	20	10, 20, 30	Decrease in biomass, increased oxidative stress indicators content (H_2_O_2_ and MDA), induced antioxidative stress responses such as flavonoids, anthocyanins, CAT, peroxidase (POD), reduced chlorophyll content, upregulation of the expression of membrane transporters and xylem cells enlargement	[24]
*Lycopersicon esculentum*	7–14	10	Lower fruit production, induction of oxidative stress, decrease in photosynthetic rate, elevated activity of antioxidant enzymes	[70]
*Arabidopsis thaliana*	10	0.2, 0.5, 3	Root growth inhibition, decreased in chlorophyll content and disruption of the thylakoid membrane structure; alteration of transcription of antioxidant and aquaporin channels	[68]
*Arabidopsis thaliana*	20	10–150	Inhibition of Arabidopsis root gravitropism, lower auxin accumulation in root tips, also, downregulation of the expression of auxin receptor-related genes	[77]
*Arabidopsis thaliana* *Populus deltoids × nigra, DN-34*	5, 10, 25	0.01–100	Toxicity of AgNPs increased with decreasing nanoparticles size; however, the stimulatory effect on fresh weight, evapotranspiration, and root elongation at sublethal concentrations	[78]
*Arabidopsis thaliana*	10	12.5	delay in flowering, decrease in petal development and vegetative growth, and pollen viability, downregulation of genes involved in floral development	[79]
*Lemna minor*	10–80	0.005–0.04	number of fronds decreased, growth reduction, chlorosis in leaves	[80]
*Cymodocea nodosa*	35	0.0002–0.2	length of leaves decreased, induction of oxidative stress indicator (H_2_O_2_), and antioxidative enzymes activity, less actin and tubulin filaments	[81]
*Zea mays (seedlings)*	49	7.5	Inhibition of root and leaf growth, induction of O_2¯_, H_2_O_2_, and MDA, increased activity of antioxidative enzymes (SOD, GR, APX),	[82]
*Vicia faba*	25, 50, 75	100	Size dependent growth decrease, leaf necrosis and damage, and stomatal conductivity decrease	[83]

**Table 2:** Overview on phytotoxicity functions of AgNPs on soil species.

Species	Size	Concentration	Major Functions	References
*Escherichia coli* and Nitrifying bacteria	16 nm	0.1–1 mg L^−1^	AgNPs inhibit respiration and nitrification (lack of change in dissolved oxygen); the effect varies depending on the size and bioavailability of the NPs.	[109]
Ammonia-oxidizing bacteria (AOB)	118 ± 11 nm	0.5–50 mg L^−1^	Silver treatments affect ammonia-oxidizing bacteria (AOB), reducing Nitrification potential rates.	[110]
*Bacillus subtilis*	27 nm	0.5–50 mg L^−1^	The growth of *B. subtilis* is affected by AgNPs depending on the size of the AgNPs which probably plays a role in toxicity differences.	[110]
*Nitrosomonas europaea*	AgNPs (35 nm)/AgNO_3_	0.075–0.75 mg L^−1^	The oxidation and production of NO_2_ by regulating the gene expression (nitric oxide reductase, ammonia monooxygenase, and nitrite reductase).	[111]
*Nitrosomonas europaea*	AgNPs (25.5 nm)/Ag^+^	0.1 mL/h of solution, 0.05–2 ppm Ag^+^, 1.5–20 ppm AgNPs	Low concentrations of AgNPs cause enzymatic inhibition (of ammonia monooxygenase enzyme) and high concentrations cause cell death.	[112]
Soil microbial community	BAM-N001 (20 nm)/AgNO_3_	0.01 mg/kg	The toxicity of AgNPs increases over time (possibly due to Ag^+^ release). AgNPs cause a decrease in biomass and the activity of soil microorganisms.	[97]
Soil bacterial phyla	BAM-N001 (20 nm)/AgNO_3_	0.01 mg/kg	Long-term exposure to AgNPs cause a decrease in several phyla of bacteria, affecting important functions of soil like nitrification or organic carbon transformation.	[113]
Bacterial and fungal assemblages	AgNPs (20 nm)/ AgMPs (3000 nm)	0.066% and 6.6%	Small particles are more toxic (cause a decrease in respiration, signature bacterial fatty acids, changes in richness and evenness in bacterial and fungal DNA sequence assemblages).	[114]
Heterotrophic bacterial and nitrifying communities	NM-300K (15 nm)	1.67 and 5 mg/kg supplied in one or three applications	Single application has a stronger effect on potential nitrification than split doses (i.e., same dose applied in 3 doses), whereas of respiration an opposite pattern is observed.	[115]
*Azotobacter vinelandii*	10 and 50 nm	2 and 10 mg L^−1^ for nano-Ag 10; 10 and 100 mg L^−1^ for nano-Ag 50	10 and 50 nm AgNPs induced apoptosis by 20.23% and 3.14%, reduced cell number, structural damage, inhibition of biological nitrogen fixation (BNF), ROS generation	[116]
*Nitrosomonas europaea* ATCC-19718	7 ± 3 (PVA doped) 40 ± 14 (Na_2_ATP doped)	1, 5 and 10 mg L^−1^	Capping and size dependent decrease in NH_3_ oxidation, cell wall damage, and disintegrated nuclei	[117]
Ammonia oxidizing microorganisms	15 nm	1, 10, and 100 µg g^−1^ dry soil	10 and 100 μg g^−1^ AgNPs significantly inhibited soil urease activity and nitrification	[118]
Soil microbial activity	20.4 and 10 nm	0.1, 1, and 10 mg kg^−1^ soil	Decreased soil microbial metabolic activity, nitrification ability, and the abundances of ammonia-oxidizing bacteria at 0.1–10 mg kg^−1^ AgNPs	[119]
Mycorrhizal clover (*Trifolium repens*)	20.6 ± 3.1 (AgNPs)	0.01–1 mg kg^−1^	Drastic decrease in biomass of mycorrhizal clover, root nutrient acquisition of AMF, and glomalin content	[120]
*Glomus aggregatum*-Faba bean	5–50	800 μg kg^−1^ sandy soil-loam mixture	Lowered mycorrhizal colonization, glomalin content, and mycorrhizal responsiveness	[121]
AMF (unspecified)-Tomato	2 and 15 nm	12–36 mg kg^−1^ soil	Dose dependent AgNPs decrease in AMF colonization	[25]
Soil microbial activity	20 nm ± 10	50 mg kg^−1^	Decrease in urease and dehydrogenase activity, bacterial and archaeal amoA gene abundance in soil	[122]
Soil microbial activity	2–50 nm (average 35 nm)	550 mg/pot	Pyrosequence analysis showed no significant effect on soil microbial richness; however, individual analysis affected bacterial groups	[123]
Soil microbial activity	15–20 nm	220 mg kg^−1^	Decrease in C and N biomass and modification of microbial community structure	[124]
Soil microbial activity	21 ± 17 nm	0.14 mg kg^−1^	AgNPs caused a modification in the bacterial community	[125]
Soil microbial activity	10 and 50 nm	1600, or 3200 µg Ag kg^−1^ dry soil	Decreases in enzymatic activities due to AgNPs	[126]
Soil microbial activity	20.08 nm ± 2.24	1–1000 mg kg^1^	AgNPs induced effects on enzymes	[99]

**Table 3:** Phytoremediation of AgNPs.

Plant	Type of NPs	Description	Effect	Reference
*Phragmites australis*	AgNPs	It accumulated silver only in roots and in leaves and stems there was no metal accumulation	Phytoremediation in estuarine areas, Phytostabilization	[49]
*Pistia stratiotes*	AgNPs	Extracted silver from source in short period of time, Easy handling and can be used in polyculture	Phytoremediation of water source	[51]
*Egeria densa*	AgNPs	Plants absorbed AgNPs at concentrations as low as 5 ppm, bioaccumulation proportional to concentration of NPs	Phytoremediation of water source	[56]
*Phanerochaete chrysosporium*	AgNPs	Uptake of NPs in presence of cysteine amino acids	Phytoremediation of aquatic source	[144]
*Zea mays*	AgNPs	NPs increase the bioremediation potential of three PGPRs isolated from municipal wastewater	Phytoremediation of municipal wastewater	[134]
*Ipomoea carnea*, *Plantago major*, *Camellia sinensis*	AgNPs	Green synthesized NPs from few medicinal plants displayed bioremediation potential	Fipronil (an insecticide) contaminated water	[145]
*Lagerstroemia speciosa*	AgNPs	methyl orange and methylene blue showing 310- and 290-min degradation time, respectively	methyl orange and methylene blue dyes	[146]
*Aloe barbedensis*, *Azadirachta indica* and *Coriandrum sativum*	AgNPs and CuNPs	Green synthesized NPs from three plants decontaminated naphthalene in water	Wastewater remediation	[147]
